# Gamma-Aminobutyric Acid Enhances Cadmium Phytoextraction by *Coreopsis grandiflora* by Remodeling the Rhizospheric Environment

**DOI:** 10.3390/plants12071484

**Published:** 2023-03-28

**Authors:** Yingqi Huang, Boqun Li, Huafang Chen, Jingxian Li, Jianchu Xu, Xiong Li

**Affiliations:** 1 Department of Economic Plants and Biotechnology, Yunnan Key Laboratory for Wild Plant Resources, Kunming Institute of Botany, Chinese Academy of Sciences, Kunming 650201, China; 2 University of Chinese Academy of Sciences, Beijing 100049, China; 3 Honghe Center for Mountain Futures, Kunming Institute of Botany, Chinese Academy of Sciences, Honghe 654400, China; 4 Science and Technology Information Center, Kunming Institute of Botany, Chinese Academy of Sciences, Kunming 650201, China

**Keywords:** gamma-aminobutyric acid, heavy metal, phytoextraction, soil amendment, plant growth-promoting rhizobacteria

## Abstract

Gamma-aminobutyric acid (GABA) significantly affects plant responses to heavy metals in hydroponics or culture media, but its corresponding effects in plant–soil systems remain unknown. In this study, different GABA dosages (0–8 g kg^−1^) were added to the rhizosphere of *Coreopsis grandiflora* grown in Cd-contaminated soils. Cd accumulation in the shoots of *C. grandiflora* was enhanced by 38.9–159.5% by GABA in a dose-dependent approach because of accelerated Cd absorption and transport. The increase in exchangeable Cd transformed from Fe-Mn oxide and carbonate-bound Cd, which may be mainly driven by decreased soil pH rather than GABA itself, could be a determining factor responsible for this phenomenon. The N, P, and K availability was affected by multiple factors under GABA treatment, which may regulate Cd accommodation and accumulation in *C. grandiflora*. The rhizospheric environment dynamics remodeled the bacterial community composition, resulting in a decline in overall bacterial diversity and richness. However, several important plant growth-promoting rhizobacteria, especially *Pseudomonas* and *Sphingomonas*, were recruited under GABA treatment to assist Cd phytoextraction in *C. grandiflora*. This study reveals that GABA as a soil amendment remodels the rhizospheric environment (e.g., soil pH and rhizobacteria) to enhance Cd phytoextraction in plant–soil systems.

## 1. Introduction

Heavy metals (HMs) are global pollutants that are highly hazardous to ecosystems and public health [1]. Plants in direct contact with HMs in soils and crops and vegetables are important sources of HMs entering human bodies via the food chain [2]. In this context, low-HM-accumulating crop and vegetable cultivars are important breeding objectives. However, plants with high HM tolerance and accumulation capabilities show potential for remediating HM-polluted soils (e.g., phytoextraction and phytostabilization) [3]. Thus, preventing HMs from entering crops/vegetables for food safety and enhancing phytoremediation efficiency is essential in addressing increasing HM pollution. Plant growth conditions (e.g., soil pH and nutrient level) and HM bioavailability [4] in soils directly determine HM uptake by plants. Therefore, regulating the soil microenvironment, which can be effectively achieved by the application of soil amendments [5], is a crucial strategy for minimizing HM uptake by crops or enhancing HM phytoremediation efficiency.

The effects of soil amendments on HM mobility in soils have attracted increasing attention over the past 20 years [6]. Soil additives, such as biochar, phosphate compounds, manure, compost, or liming materials, can reduce HM bioavailability in soils via various mechanisms [6], whereas soil chelators can enhance phytoremediation efficiency by activating HM bioavailability, stimulating the features and vitality of plant roots, and/or increasing the HM bearing capacity of plants [7,8]. However, many soil amendments have several challenges, including actual amendment effects, environmental interference resistance, and environmental friendliness [7], that hinder their large-scale application for HM pollution remediation in soils. Hence, efficient and environmentally friendly soil amendments such as natural amino acids and aminopolycarboxylic acids [9,10] are worth exploring in the future.

Gamma-aminobutyric acid (GABA), a natural non-protein amino acid, is widely found in organisms. GABA displays great application potential in various areas because of its environmentally-friendly and mass-producible features [11]. GABA-mediated HM detoxification in plants has been frequently reported [12,13,14,15,16]. Several studies have also found that exogenous GABA can affect plant HMs’ and metalloids’ uptake under hydroponic or artificial-nutrition conditions. For instance, GABA decreased Cr accumulation in *Brassica juncea* in nutrient solutions [15], As accumulation in rice in nutrient media [13], Cd accumulation in maize in perlite and cocopite [16], and Cd uptake in apple seedlings in nutrient solutions [14]. However, little is known about the effects of GABA on HM tolerance and uptake by plants in soils. This is an intriguing topic because GABA may regulate the uptake of HMs by plants by interacting with multiple soil environmental factors (especially the microbial community). In this study, the potential effects and rhizospheric mechanisms of exogenous GABA on Cd phytoextraction efficiency of a Cd accumulator, *Coreopsis grandiflora* [17], were explored. The study aims to provide novel perspectives for artificially regulating HM transport in plant–soil systems from interactions between GABA and soil factors (e.g., rhizobacteria).

## 2. Results

### 2.1. Effects of GABA Addition on the Growth and Cd Uptake of C. grandiflora

**Figure 1:**
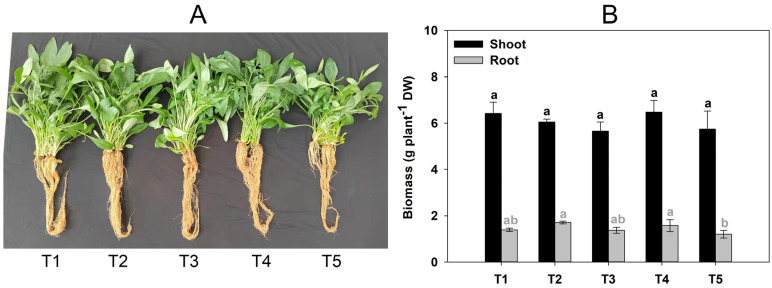

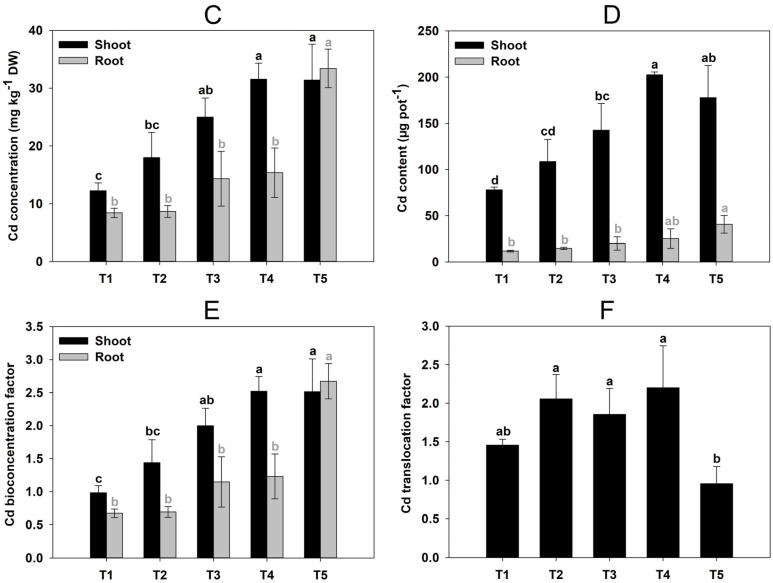
Growth and Cd accumulation characteristics of *C. grandiflora* in Cd-contaminated soils supplemented with 0 (T1), 1 (T2), 2 (T3), 4 (T4), and 8 (T5) g kg^−1^ gamma-aminobutyric acid (GABA). (**A**) Plant morphological features at harvest. (**B**) Plant biomasses (ANOVA for shoot: F = 1.109, P = 0.404, degree of freedom = 4; ANOVA for root: F = 3.140, *p* = 0.065, degree of freedom = 4). (**C**) Cd concentrations in plants (ANOVA for shoot: F = 9.143, *p* = 0.002, degree of freedom = 4; ANOVA for root: F = 19.554, *p* = 0.000, degree of freedom = 4). (**D**) Cd contents accumulated in plants in a single pot (ANOVA for shoot: F = 9.707, *p* = 0.002, degree of freedom = 4; ANOVA for root: F = 5.131, *p* = 0.016, degree of freedom = 4). (**E**) Cd bioconcentration factors (ANOVA for shoot: F = 9.049, *p* = 0.002, degree of freedom = 4; ANOVA for root: F = 19.675, *p* = 0.000, degree of freedom = 4). (**F**) Cd translocation factors (ANOVA: F = 4.468, *p* = 0.025, degree of freedom = 4). Data represent means ± standard deviations (**B**–**F**: n = 3); the same-colored bars labelled with different letters (a, b, c, and d) indicate significant differences (*p* < 0.05, Duncan’s test, one-way ANOVA) between groups. DW: dry weight.

Compared to the control (T1) samples, GABA supplementation had insignificant effect on the growth of *C. grandiflora* (Figure 1A,B). However, the average Cd concentrations in the shoots (12.27–31.40 mg kg^−1^) and roots (8.43–33.43 mg kg^−1^) of *C. grandiflora* indicated an increasing trend with GABA supplementation in a dose-dependent manner (Figure 1C). Total Cd content increased by 38.9%, 82.7%, 159.5%, and 127.9% in shoots and 25.9%, 70.4%, 115.3%, and 247.4% in roots when the plants were treated with 1 (T2), 2 (T3), 4 (T4), and 8 g kg^−1^ (T5) GABA, respectively, compared with the control group (T1) (Figure 1D). The change in Cd BCFs (Figure 1E) was the same as that of Cd concentrations (Figure 1C) in plants, but Cd TFs were not significantly affected by GABA addition (Figure 1F). These results suggest that GABA added to soils effectively promoted Cd accumulation in *C. grandiflora* but had a poor effect on Cd transport rates from roots to shoots.

### 2.2. Effects of GABA Addition on Cd Bioavailability in the C. grandiflora Rhizosphere

Total Cd concentrations remained similar in different soils, but the concentrations of different Cd fractions were remolded with GABA supplementation (Table 1). Exchangeable Cd concentrations in the T3 and T5 soils were significantly higher (*p* < 0.05) than those in the T1 soil, whereas Fe-Mn oxide Cd concentrations showed a significant decrease (*p* < 0.05) in the T3 and T5 soils compared to the T1 soil (Table 1). In contrast, carbonate-bound Cd concentration was also significantly lower (*p* < 0.05) in the T5 soil than in the T1 soil (Table 1). The organic-bound and residual Cd concentrations showed no differences between the different soils (Table 1). These results indicate that GABA supplementation drives the transformation process from Fe-Mn oxide and carbonate-bound Cd to exchangeable Cd in the rhizosphere of *C. grandiflora*, resulting in an increase in Cd bioavailability.

### 2.3. Leaching of Cd in Soils by GABA

Following leaching, total Cd concentrations in both the residual soils and filtrates were similar among the different treatments (Supplementary Figures S1 and S2), indicating that GABA did not directly increase water-soluble Cd in the soils.

### 2.4. Effects of GABA Addition on Physicochemical Indices in the C. grandiflora Rhizosphere

**Figure 2:**
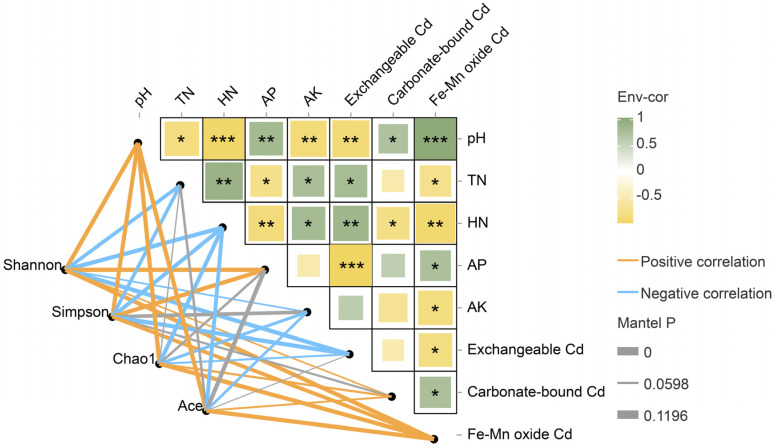
Correlation network between soil physicochemical indices and alpha diversity indices. The color block size in the correlation heatmap indicates the absolute value of the correlation coefficient. *, **, and *** represent 0.01 < *p* < 0.05, 0.001 < *p* < 0.01, and *p* < 0.001, respectively. Orange and blue network lines indicate significantly positive and negative correlations (*p* < 0.05), respectively.

The pH values showed a decreasing trend (*p* < 0.05) with increasing doses of GABA supplementation (Table 2). Insignificant differences in the concentrations of OM, TP, and TK were found between different soils, whereas an increasing trend was observed in TN concentration with GABA supplementation (Table 2). HN and AK concentrations were significantly (*p* < 0.05) higher in the T5 soil than in the T1 and T3 soils, whereas AP concentrations in the T3 and T5 soils were significantly (*p* < 0.05) decreased with GABA supplementation compared to the T1 soil (Table 2). These altered soil physicochemical indices showed multiple correlations (Figure 2), indicating complex interactions among them.

### 2.5. Bacterial Community Composition in the C. grandiflora Rhizosphere

#### 2.5.1. Composition of Bacterial Communities

**Figure 3:**
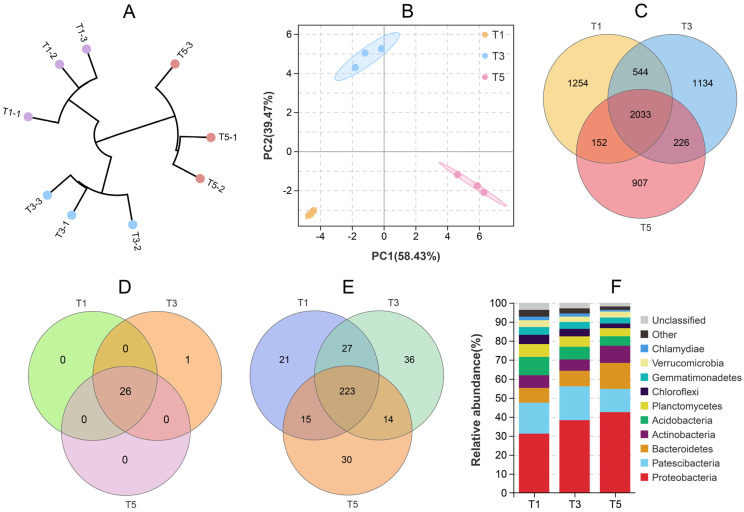
The rhizosphere bacterial community composition of *C. grandiflora* grown in Cd-contaminated soils supplemented with 0 (T1), 2 (T3), and 8 (T5) g kg^−1^ GABA. The unweighted pair group method with arithmetic mean clustering tree (**A**) and principal component analysis (**B**) of samples at the operational taxonomic unit (OTU) level. (**C**) Venn diagram of OTUs among different soils. (**D**) Venn diagram of bacterial phyla among different soils. (**E**) Venn diagram of bacterial genera among different soils. (**F**) Stacked diagram showing the relative abundance of the top ten bacterial phyla in different soils.

The sequencing results showed that 120,868–134,136 raw reads were generated for different samples (Supplementary Table S2). The average numbers of bacterial operational taxonomic units (OTUs) identified in the T1, T3, and T5 soils were 4791, 4747, and 4042, respectively, in which the T5 soil was significantly lower than those in the T1 and T3 soils (Supplementary Table S2). The unweighted pair group method with arithmetic mean (UPGMA) clustering tree showed that samples from the same group clustered together, and different groups were clearly separated (Figure 3A). Principal component analysis (PCA) also showed similar results (Figure 3B). These results suggest that the bacterial community composition in the rhizosphere of *C. grandiflora* varied between different treatments.

The alpha diversity of bacterial communities showed significant differences among the different soils (Supplementary Table S3). The Shannon and Simpson indices were significantly (*p* < 0.05) reduced with GABA supplementation in a dose-dependent manner, while the Chao1 and Ace indices in the T5 soil were significantly (*p* < 0.05) lower than those in the T1 and T3 soils (Supplementary Table S3). These results suggest that GABA supplementation decreased the total bacterial richness and diversity in the *C. grandiflora* rhizosphere (Supplementary Table S3). The correlation network analysis indicated that many soil physicochemical indices were significantly (*p* < 0.05) correlated with the four alpha indices (Figure 2), suggesting that these soil physicochemical indices may affect bacterial community composition.

There were 2033 common OTUs identified in the T1, T3 and T5 soils, accounting for 42.4–50.3% of the total OTUs (Figure 3C). The unique bacterial OTU numbers showed the following trend: T1 (1254) > T3 (1134) > T5 (907) (Figure 3C). At the phylum level, the three soils shared 26 bacterial phyla, and only one unique bacterial phylum was identified in the T3 soil (Figure 3D). Similarly, the three soils shared the majority of the bacterial genera (223), and 21–36 specific bacterial genera were identified in different soils (Figure 3E). The top bacterial phyla (21) and genera (98) with relatively high abundance (>0.1%) are shown in Supplementary Tables S4 and S5, respectively. Among them, Proteobacteria (31.2–42.5%), Patescibacteria (12.3–17.9%), Bacteroidetes (7.8–13.6%), Actinobacteria (6.0–9.0%), and Acidobacteria (5.0–9.6%) were the dominant phyla (Figure 3F).

#### 2.5.2. Variations in Rhizobacteria under GABA Addition

**Figure 4:**
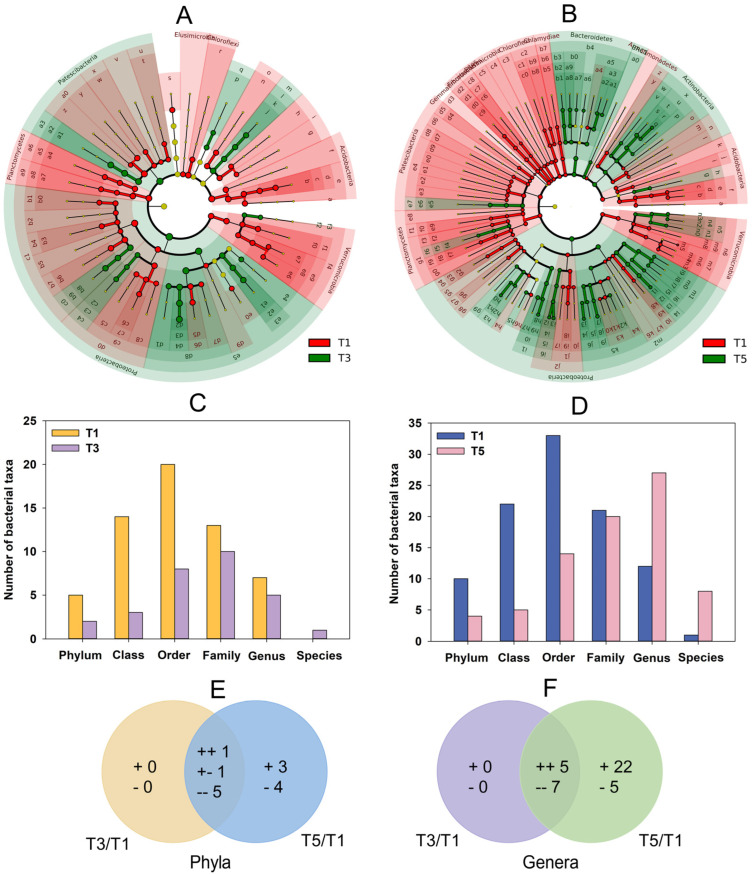
Linear discriminant analysis (LDA) and effect size analysis (LDA scores > 3) showing the indicator bacteria in rhizosphere soils of *C. grandiflora* supplemented with 0 (T1), 2 (T3) and 8 (T5) g kg^−1^ GABA. (**A**) Cladogram showing dominant bacteria between T1 and T3 soils. Identifiers labelled on the cladogram correspond to those in Supplementary Table S6. (**B**) Cladogram showing dominant bacteria between T1 and T5 soils. Identifiers labelled on the cladogram correspond to those in Supplementary Table S7. (**C**) Numbers of dominant bacteria at different taxonomic levels between T1 and T3 soils. (**D**) Numbers of dominant bacteria at different taxonomic levels between T1 and T5 soils. (**E**) Venn diagram of the differential bacterial phyla between T3 and T5 soils compared to the T1 soil. (**F**) Venn diagram of the differential bacterial genera between T3 and T5 soils compared to the T1 soil.

Changes in abundance, which were performed using linear discriminant analysis (LDA) effect size analysis (LDA scores > 3), were identified for many bacterial taxa in the T3 and T5 soils compared to those in the T1 soil (Figure 4A,B; Supplementary Tables S6 and S7). Generally, more bacterial taxa at each taxonomic level were induced or declined in the T5 soil than in the T3 soil (Figure 4C,D). A total of seven common bacterial phyla changed relatively consistently in both the T3 and T5 soils, and another seven bacterial phyla showed a specific change in the T5 soil (Figure 4E). Similarly, 12 common bacterial genera changed consistently in both the T3 and T5 soils, and another 27 bacterial genera were specifically altered in the T5 soil (Figure 4F). These results indicated that GABA had a significant effect on the variations in rhizobacteria in a dose-dependent manner.

**Figure 5:**
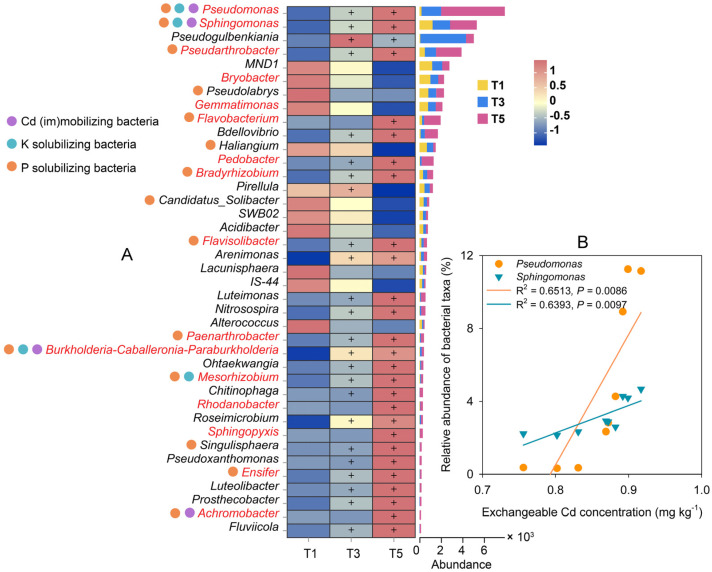
Dynamic heatmap and stacked graph showing the enriched bacterial genera (**A**) and correlation analysis between relative abundance of bacterial taxa and soil exchangeable Cd concentration (**B**) in *C. grandiflora* rhizosphere soils supplemented with 2 (T3) and 8 (T5) g kg^−1^ GABA compared with the control soil (T1). The relative abundances of bacterial genera in the graph are normalized at the row level of the heatmap. The plus (+) sign indicates the rhizobacteria enriched in the T3 and/or T5 soils. Bacterial genera in red font indicate plant growth-promoting rhizobacteria, and bacterial genera marked with purple, green, and orange circles indicate rhizobacteria involved in Cd mobilization/immobilization, K solubilization, and P solubilization, respectively.

At the genus level, many rhizobacteria that increased were plant growth-promoting rhizobacteria (PGPR), and some of them were involved in Cd (im)mobilization and/or P and K solubilization (Figure 5A). In particular, relative abundance dynamics of the two dominant genera, *Pseudomonas* and *Sphingomonas*, showed significant correlations with changes in exchangeable Cd concentrations in soils (Figure 5B), indicating that these bacterial taxa may be the driving factors for the transformation of Cd chemical fractions.

## 3. Discussion

This study showed that GABA supplementation to the root zone improved Cd accumulation in *C. grandiflora* (Figure 1C,D), which indicated that GABA acts as a soil amendment. The results were different from previous findings that foliar spraying of exogenous GABA [18] and its addition in hydroponics [14,15], semihydroponics [16], or nutrient media [13] reduced HM uptake by plants. A similar phenomenon has also been observed for cysteine, for example, which increased Cd accumulation in *Solanum nigrum* in soils [19] and Hg accumulation in *Arabidopsis* in Hoagland nutrient solution [20] but reduced Cr uptake in *B. napus* in MS media [21] and in maize shoots in Hoagland nutrient solution [22]. These results indicate that cysteine did not produce a consistent effect on different HM-plant systems even in the similar environmental media [20,22]. Accordingly, the effects of GABA on HM uptake by plants in different soil–HM–plant systems are also mutable and should attract further research. The distinct effects of GABA on HM uptake by plants in soils and nutrient solutions/media may be attributed to the more complex microenvironments in soils, including HM speciation, soil properties, and microbial community composition.

Cd speciation and bioavailability in the *C. grandiflora* rhizosphere were analyzed because they determine HM uptake by plants [23]. Interestingly, in this study, GABA addition improved Cd bioavailability in the rhizosphere of *C. grandiflora* by facilitating the transformation of Fe-Mn oxide and carbonate-bound Cd into exchangeable Cd (Table 1). The exchangeable fraction of HMs easily migrate in soils and is readily absorbed by plants, whereas the Fe-Mn oxide fraction of HMs is not readily available [24]. However, Cd leaching results (Supplementary Figures S1 and S2) showed that GABA could hardly activate Cd bioavailability directly in soils like other soil chelators [25].

Soil physicochemical indices and microbial community composition in the *C. grandiflora* rhizosphere were thus analyzed to explore the mechanisms of GABA. Significant changes in soil pH and TN, HN, AP, and AK concentrations were observed (Table 2), indicating that GABA addition remodeled rhizospheric microenvironments. Decreased soil pH, which may be due to changes in the proportion of anions and cations across the rhizosphere or organic acids secreted by plants, should be a key factor affecting the transformation of Cd speciation in this study according to previous reports [26,27]. Soil pH may also be a driving factor [28,29,30] for the changes in N, P, and K availability (Figure 2). Additionally, GABA could serve as a soil N source, contributing to an increase in the concentrations of both TN and HN (Table 2). Previous studies reported that the availability of N, P, and K affects plant growth and HM uptake [31,32], indicating a complex interaction between nutrient transformation and Cd absorption in the rhizosphere of *C. grandiflora* under GABA addition.

The reassembling of bacterial community composition was also observed in the rhizosphere of *C. grandiflora* under GABA addition (Figure 3 and Figure 4), which can largely affect Cd bioavailability in soils and Cd uptake by plants [10]. The overall bacterial diversity and richness in the *C. grandiflora* rhizosphere was significantly diminished (*p* < 0.05) under GABA addition (Supplementary Table S3). At each classification level, the abundance of some bacterial taxa was markedly increased or decreased with GABA supplementation (Figure 4; Supplementary Tables S6 and S7). The results were supported by previous studies showing that GABA, as a root exudate, altered the microbiome composition throughout the root systems of rice, wheat, and maize [33,34]. According to the correlation network analysis results (Figure 2), the dynamics of bacterial community composition should be attributed to multiple soil factors and not to GABA solely. Like other amino acids, GABA is a ready nutrition source for many microorganisms, leading to the proliferation of some bacteria. It may also serve as a signal molecule luring the chemotaxis and colonization of some rhizobacteria [35]. Moreover, the acidified soil environment and altered bioavailability of some elements (e.g., Cd, N, P and K) may also be partially responsible for the changes in rhizobacteria [36,37]. In addition, GABA and these altered soil physicochemical indices likely reprogramed root exudates, which ultimately interplay with rhizospheric microbiota [38].

Many potential PGPR [39,40] were upregulated with GABA supplementation (Figure 5A), confirming the environmental friendliness of GABA. These PGPR can improve *C. grandiflora* resistance to Cd stress through various mechanisms, such as promoting nutrient acquisition, producing growth regulators, or resisting pathogenic bacteria [39]. Moreover, some PGPR (Figure 5A) can also regulate Cd uptake by plants by affecting Cd mobility and/or ameliorating soil microenvironments [39,41], as well as regulating the expression of metal transporters in plants [42]. The increase in several PGPR, such as *Pseudomonas*, *Sphingomonas*, and *Burkholderia-Caballeronia-Paraburkholderia* (Figure 5A), affects Cd mobilization and K solubilization [39,43], in accordance with the increase in the bioavailability of Cd and K (Table 1 and Table 2). Although many P-solubilizing rhizobacteria were upregulated under GABA supplementation (Figure 5A), a decrease in AP concentration was observed (Table 2) in the *C. grandiflora* rhizosphere, which can be attributed to trade-offs among multiple processes of P solubilization, insolubilization, and intake by plants.

In particular, the two dominant PGPR (i.e., *Pseudomonas* and *Sphingomonas*) of the Proteobacteria phylum (Figure 5A) may play important roles in regulating Cd phytoextraction because their abundances showed significantly positive correlation with soil exchangeable Cd concentrations (Figure 5B). *Pseudomonas* spp. are known to have diverse plant beneficial traits and to improve plant health and vitality under HM stress [44]. Many *Pseudomonas* strains mediate Cd absorption and transport, promoting Cd phytoextraction efficiency. For example, inoculation of two *P. aeruginosa* strains (ZGKD2 and ZGKD5) improved Cd phytoextraction from *S. nigrum* by increasing Cd concentration and translocation from roots to shoots [45]. *Sphingomonas* spp. are known as the main components of the root-associated bacterial taxa that undergo HM (especially Cd and Zn) phytoextraction [41,46]. *Pseudomonas* and *Sphingomonas* spp. enrichment in the rhizosphere of *C. grandiflora* contributed crucially to the promotion of Cd phytoextraction. In addition, many *Pseudomonas* spp. and some *Sphingomonas* spp. have been identified as P- and K-solubilizing bacteria [43,47,48], indicating that these two bacterial taxa also participate in the regulation of the nutrient cycles in the rhizosphere of *C. grandiflora*.

## 4. Materials and Methods

### 4.1. Experimental Treatment

The soil was obtained via a half-and-half mixture by volume proportion of the locally cultivated soil in Kunming and previously Cd-contaminated soil in the laboratory [49]. The soils were sieved, fully mixed, and loaded into uniform flowerpots (h = 17.5 cm, d = 18.5 cm) in equal aliquots, which were then placed in a glass greenhouse (day: approximately 82% natural light, 12–14 h, 23–25 °C; night: 10–12 h, 18–20 °C; humidity: 40–60%) to equilibrate for a week. The basic parameters of the homogeneous soil are provided in Supplementary Table S1.

*C. grandiflora* seeds were surface-sterilized (1% NaClO solution, 10 min), sown in the aforementioned pots, and thinned to three seedlings per pot as soon as the seeds germinated. After growing for approximately one month, five groups of plants were separately supplemented with 0 (T1), 1 (T2), 2 (T3), 4 (T4), and 8 (T5) g kg^−1^ GABA in rhizospheric soils. GABA was supplemented to the soil samples four times every five days according to a previously described method [10]. For each supplementation, one-fourth of solid GABA (Macklin, Shanghai, China) for the targeted dosages per pot was dissolved in 150 mL of deionized water and poured evenly around the plant roots. The experiment ended one month after the fourth GABA supplementation. Three biological replicates were prepared for each treatment.

### 4.2. Sample Collection and Biomass Measurement

After the experiment, shoots and roots of *C. grandiflora* plants were collected separately and the roots were cleaned using Na_2_EDTA solution (15 mM, 20 min) to remove Cd^2+^ adsorbed on the root surface [50]. Plant samples were oven dried (80 °C, 48 h) for biomass measurement. Rhizospheric soils that naturally adhered to the root systems after gentle shaking [10,17] were collected for subsequent measurements.

### 4.3. Cd Accumulation Characteristics Analysis in Plants

Total Cd concentrations in the dried shoots and roots were determined using the method described in the Supplementary Methods. The Cd bioconcentration factor (BCF), translocation factor (TF), and total accumulation content in the *C. grandiflora* shoots and roots were calculated using previously reported formulas [51,52].

### 4.4. Cd Speciation Determination in Soil

Total Cd and the different Cd fractions (i.e., exchangeable Cd, Fe-Mn oxide Cd, organic-bound Cd, carbonate-bound Cd, and residual Cd) that were extracted using a Tessier sequential extraction procedure [24,27] in rhizospheric soils were determined using a graphite furnace atomic absorption spectrometer (GFAAS) (ZEEnit700P, Analytik Jena, Jena, Germany).

### 4.5. Leaching Experiment

The leaching of GABA on Cd in the soils, used to investigate whether exogenous GABA can directly mobilize Cd in soils, was performed according to previously reported methods [53,54,55]. Concentration gradients of GABA solutions (i.e., 0, 1, and 4 g L^−1^) were prepared as eluents in accordance with the GABA concentration range added to the soils (see Section 4.1). Cd-contaminated soil samples (2.0 g) were loaded in 50 mL centrifuge tubes, and 20 mL eluents were added. The samples were shaken (200 rpm, 25 °C, 24 h) and then centrifuged (1800× *g*, 10 min) using a high-speed tabletop centrifuge (Eppendorf 5810R, Hamburg, Germany). The supernatants were filtered through microporous membranes (0.45 μm), and the residual soils were oven dried (80 °C, 48 h). Cd concentrations were detected in both filtrates and residual soils using GFAAS. Each treatment was repeated three times.

### 4.6. Determination of Soil Physicochemical Indices

Soil physicochemical indices, including pH, organic matter (OM), and total N (TN), K (TK), P (TP), hydrolysable N (HN), and available K (AK) and P (AP) concentrations, were determined using the methods in the corresponding Chinese testing standards [10,56,57]. The detailed methods for each determination are provided in the Supplementary Methods.

### 4.7. Soil Bacterial Community Analysis

DNA extraction, 16S rDNA amplification, sequencing, and bioinformatics analyses for soil bacterial community analysis were performed using previously described methods [49]. The V3–V4 region of the 16S rDNA gene was amplified by PCR for Illumina Novaseq 6000 sequencing using the 341F (5′−CCTACGGGNGGCWGCAG−3′) and 806R (5′−GGACTACHVGGGTATCTAAT−3′) primer pairs. The raw sequencing reads were deposited in the Science Data Bank (DOI: 10.57760/sciencedb.03180).

### 4.8. Statistical Analysis

Significance analysis among groups (n ≥ 3) was performed using one-way ANOVA with Duncan’s multiple range tests via SPSS 26.0 (IBM, Amunk, NY, USA). Linear regression analysis was performed using SigmaPlot 10.0 (Systat, San Jose, CA, USA). Correlation network analysis between soil indices and bacterial alpha indices was performed using the online Omicsmart platform (http://www.omicsmart.com; accessed on 8 October 2022).

## 5. Conclusions

This study established that GABA acted as a soil amendment to effectively enhance Cd phytoextraction by *C. grandiflora*. Complex interactions between soil factors can be used to explain this result. The increase in exchangeable Cd transformed from Fe-Mn oxide and carbonate-bound Cd was a determining factor responsible for enhancing Cd phytoextraction. The decreased soil pH rather than GABA itself should be an important driving factor for this process. The improved HN and AK concentrations may affect Cd resistance and accumulation in *C. grandiflora*. The remolded rhizospheric microenvironments had a significant impact on the bacterial community composition in the *C. grandiflora* rhizosphere. Interestingly, the upregulation of several important PGPR under GABA addition, especially *Pseudomonas* and *Sphingomonas*, may play important roles in assisting Cd phytoextraction in *C. grandiflora* through various mechanisms. These findings suggest combined GABA-PGPR strategies for enhancing Cd phytoextraction and improving our understanding of the effects of GABA as a root exudate on the interaction between HMs and plants. Several intriguing questions from this study deserve further exploration. For instance, the similarities and differences of the effects and mechanisms of GABA supplementation in different plant–soil systems require elucidation. Moreover, sufficient verification experiments are required to determine whether GABA supplementation directly or indirectly remodels the rhizospheric microenvironment (e.g., soil pH and microbial community composition).

## Figures and Tables

**Table 1:** Cd speciation (mg kg^−1^) in the rhizosphere of *C. grandiflora* supplemented with 0 (T1), 2 (T3), and 8 (T5) g kg^−1^ gamma-aminobutyric acid (GABA).

Cd Chemsical Fractions	T1	T3	T5	ANOVA		
				F Value	*p* Value	DF
Total Cd	12.26 ± 0.53 a	12.40 ± 0.67 a	11.97 ± 0.76 a	0.224	0.805	2
Exchangeable Cd	0.80 ± 0.03 b	0.87 ± 0.01 a	0.90 ± 0.01 a	16.610	0.004	2
Carbonate-bound Cd	0.07 ± 0.01 a	0.07 ± 0.02 a	0.04 ± 0.01 b	3.452	0.101	2
Fe-Mn oxide Cd	0.25 ± 0.01 a	0.24 ± 0.01 b	0.18 ± 0.00 c	90.799	0.000	2
Organic-bound Cd	0.04 ± 0.00 a	0.04 ± 0.00 a	0.03 ± 0.00 a	0.853	0.472	2
Residual Cd	11.11 ± 0.54 a	11.18 ± 0.67 a	10.81 ± 0.76 a	0.172	0.846	2

Data represent the means ± standard deviations (n = 3); data in the same row labelled with different letters (a, b, and c) indicate significant differences (*p* < 0.05, Duncan’s test, one-way ANOVA) among different treatments. DF: degree of freedom.

**Table 2:** Soil physicochemical indices in the rhizosphere of *C. grandiflora* supplemented with 0 (T1), 2 (T3), and 8 (T5) g kg^−1^ GABA.

Soil Indices	Unit	T1	T3	T5	ANOVA		
					F Value	*p* Value	DF
pH	/	6.01 ± 0.06 a	5.75 ± 0.02 b	5.11 ± 0.04 c	238.888	0.000	2
OM	g kg^−1^	220.03 ± 8.49 a	205.97 ± 11.61 a	217.57 ± 14.13 a	0.832	0.480	2
TN	g kg^−1^	6.09 ± 0.21 b	6.43 ± 0.28 ab	6.77 ± 0.04 a	5.617	0.042	2
TP	g kg^−1^	1.41 ± 0.02 a	1.44 ± 0.02 a	1.41 ± 0.04 a	0.672	0.545	2
TK	g kg^−1^	9.38 ± 0.12 a	8.82 ± 0.48 a	9.02 ± 0.36 a	1.276	0.345	2
HN	mg kg^−1^	342.94 ± 20.13 b	366.22 ± 7.33 b	417.39 ± 6.34 a	17.435	0.003	2
AP	mg kg^−1^	29.56 ± 0.77 a	25.85 ± 0.62 b	25.06 ± 0.46 b	29.329	0.001	2
AK	mg kg^−1^	55.80 ± 2.67 b	57.50 ± 3.83 b	77.63 ± 10.47 a	6.733	0.029	2

Data represent the means ± standard deviations (n = 3); data in the same row labelled with different letters (a, b, and c) indicate significant differences (*p* < 0.05, Duncan’s test, one-way ANOVA) among different treatments. AK: available K; AP: available P; DF: degree of freedom; HN: hydrolysable N; OM: organic matter; TK: total K; TN: total N; TP: total P.

## Data Availability

Not applicable.

## Supplementary Materials

The following supporting information can be downloaded at: https://www.mdpi.com/article/10.3390/plants12071484/s1, Supplementary Methods; Figure S1: Cd concentrations in residual soils after leaching with 0, 1, and 4 g L^−1^ GABA; Figure S2: Cd concentrations in filtrates after leaching with 0, 1, and 4 g L^−1^ GABA; Table S1: Physicochemical parameters of the homogenized soil; Table S2: Basic information of 16S rDNA sequencing results; Table S3: Alpha diversity index of bacterial community; Table S4: Relative abundance (%) of the top 21 bacterial phyla in cadmium-contaminated soils treated with different GABA concentrations; Table S5: Relative abundance (%) of the top 98 bacterial genera in cadmium-contaminated soils treated with different GABA concentrations; Table S6: The indicator bacteria with linear discriminant analysis (LDA) score > 3 between T1 and T3 soils; Table S7: The indicator bacteria with linear discriminant analysis (LDA) score > 3 between T1 and T5 soils.
