# Supplementary material for: Gamma-Aminobutyric Acid Enhances Cadmium Phytoextraction by Coreopsis grandiflora by Remodeling the Rhizospheric Environment
Source: Plants (Basel). 2023 Mar 28;12(7):1484. doi: 10.3390/plants12071484 (PMC10096890; doi:10.3390/plants12071484)
Supplement: Supplementary file 1 [file plants-12-01484-s001.zip › Supplementary Figures.pdf]

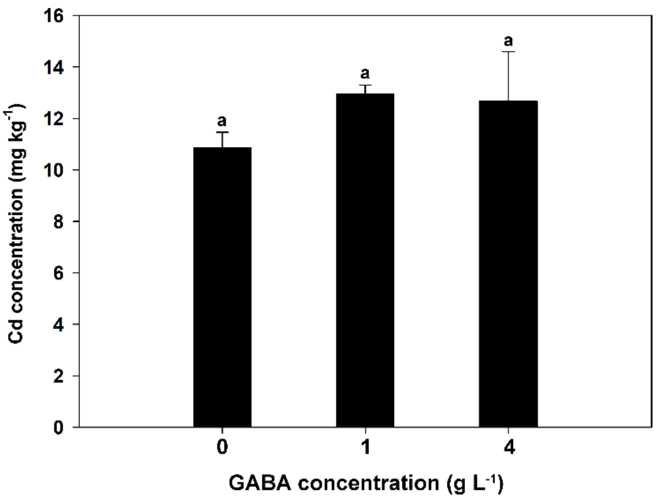

Figure S1. Cd concentrations in residual soils after leaching with 0, 1, and 4 g L^{-1} GABA. Bars labelled with the same letter (a) indicate no significant difference ($p > 0.05$, Duncan's test, one-way ANOVA) between groups.

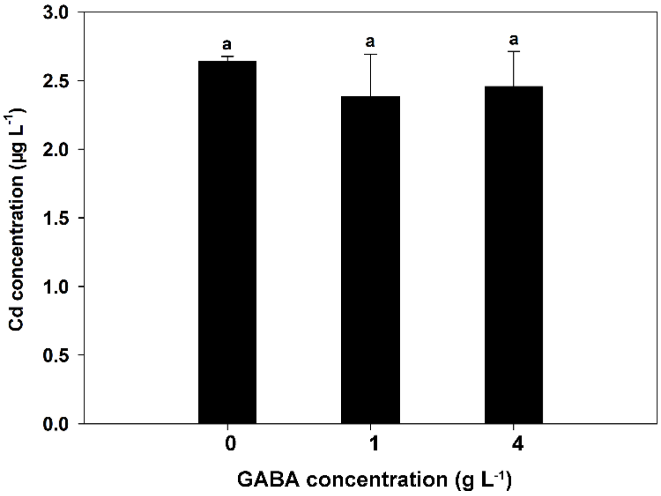

Figure S2. Cd concentrations in filtrates after leaching with 0, 1, and 4 g L^{-1} GABA. Bars labelled with the same letter (a) indicate no significant difference ($p > 0.05$, Duncan's test, one-way ANOVA) between groups.
