# Supplementary material for: Gamma-Aminobutyric Acid Enhances Cadmium Phytoextraction by Coreopsis grandiflora by Remodeling the Rhizospheric Environment
Source: Plants (Basel). 2023 Mar 28;12(7):1484. doi: 10.3390/plants12071484 (PMC10096890; doi:10.3390/plants12071484)
Supplement: Supplementary file 1 [file plants-12-01484-s001.zip › Supplementary Methods.pdf]

Detection of Cd concentration in plants

In brief, approximately 0.2–1.0 g dried samples were added in polytetrafluoroethylene digestion tanks and 5 mL of HNO_3 was injected. The tanks were left standing until the reactions were finished. The tanks were then sealed with caps and put into a microwave digestion instrument (WX-8000, Yi Yao Instrument, Shanghai, China) using the following digestion procedure: 100 ℃, 3 min; 140 ℃, 3 min; 160 ℃, 3 min; 180 ℃, 3 min; and 190 ℃, 15 min. When the temperature cooled below 50 ℃, the digestion tanks were taken to the fume hood. The digestion solutions were transferred to 50-mL volumetric flasks. The tanks were rinsed thrice using ultrapure water and the volumes were fixed to the measurement scale. The blank control was treated with the same process. Sample solutions were detected using an inductively coupled plasma mass spectrometer (ICP-MS, Thermo Fisher Scientific, USA) and the Cd concentrations were calculated according to the standard curve.

Determination of soil physicochemical indices

Soil pH determination

In brief, approximately 10 g soil samples were mixed with 25 mL KCl solutions (1 M), shocked severely for 5 min, and then remained still for 1 h at room temperature. Soil pH was detected using a pH meter.

Organic matter determination

In brief, approximately 0.5 g soil samples were mixed with 10 mL $K_2Cr_2O_7$-H_2SO_4 solutions (0.4 M) in hard test tubes. The test tubes were heated to 170−180 ℃ for 5 min in an oil bath pan. After cooling, the mixtures were transferred completely to 250 mL flasks and sized to 50 mL. The solutions were added with 3 drops of o-phenanthroline indicator and then titrated with $FeSO_4$ standard solutions (0.1 M) to brownish red. Soil samples without digestion were used for the control test. The organic matter concentrations were calculated using the following formula:

$$\text{OM} = \frac{0.1 \times (V_0 - V) \times 0.003 \times 1.724 \times 1.10}{m} \times 1000$$

OM: organic matter concentration (g kg^{-1}); V_0: volume of $FeSO_4$ standard solution for the

control test (mL); V: volume of $FeSO_4$ standard solution for the sample test (mL); m: sample weight (g).

Total N determination

In brief, approximately 1.0 g soil samples were mixed with 1.0 mL $KMnO_4$ solutions (50 g L^{-1}) and 2.0 mL concentrated H_2SO_4 for 5 min in digestion tubes. The mixtures were added with a drop of octanol and 0.5 g of reduced Fe powder. After the reaction, the digestion tubes were heated on a digestion stove for 45 min. After cooling, the mixtures were added with 2 g accelerators and 5 mL H_2SO_4 and heated on a digestion stove to yellowish green. The digestion tubes were added with 20 mL NaOH solutions (10 M) for distillation. The distillates, which were pre added with 10 mL boric acid indicators, were titrated with 0.02 M HCl standard solutions. The total N concentrations were calculated using the following formula:

$$TN = \frac{0.02 \times (V_0 - V) \times 0.014}{m} \times 1000$$

TN: total N concentration (g kg^{-1}); V_0: volume of HCl standard solution for the control test (mL); V: volume of HCl standard solution for the sample test (mL); m: sample weight (g).

Total P determination

In brief, approximately 0.25 g soil samples were added with 3 drops of alcohol and 2 g NaOH in nickel crucibles. The crucibles were heated in a high-temperature electric furnace (400 ℃, 15 min; 720 ℃, 15 min). After cooling, the samples were added with 10 mL water (80 ℃) and then transferred to 100 ml volumetric flasks. The mixtures were added with 10 mL concentrated H_2SO_4 (3 M) and sized to 100 mL using water. Approximately 2−10 mL pilot samples were transferred into 50 mL volumetric flasks, diluted to approximately 30 mL using water, and then added with 3 drops of dinitrophenol indicator. The solutions were adjusted to faint yellow using 10% Na_2CO_3 solutions, added with 5 mL molybdenum antimony antichromogenic agents, and then mixed and sized to 50 mL. The samples remained still for 30 min at room temperature and were detected using a spectrophotometer at 700 nm. Gradients of 0, 0.2, 0.4, 0.6, 0.8, and 1.0 mg L^{-1} P standard solutions were used to draw the standard curve. The total P concentrations were calculated using the following formula:

$$TP = C \times \frac{100\,mL}{m} \times \frac{50\,mL}{V}$$

TP: total P concentration (g kg^{-1}); C: P concentration in pilot sample solution obtained from the standard curve (mg mL^{-1}); V: volume of pilot sample solution (mL); m: sample weight (g).

Total K determination

In brief, approximately 0.1 g soil samples were added with 3 mL H_3NO_3 and 0.5 mL $HClO_4$ in tetrafluoroacetic acid crucibles. The crucibles were heated in an electric sand bath until the samples became paste. After cooling, the samples were added with 5 mL oxyhydrogen acid and 0.5 mL $HClO_4$ and then heated (200−225 ℃) in an electric sand bath until the samples stopped emitting white smoke. After cooling, the samples were added with 10 mL HCl and heated until the residue dissolved. After cooling, the samples were added with 2 mL 2% boric acid solutions, transferred into volumetric flasks, and sized to 100 mL using water. Gradients of 0, 1, 2, 4, 6, 8, and 10 mg L^{-1} K standard solutions were used to draw the standard curve. Appropriate pilot samples were transferred into volumetric flasks, added with NaCl solution (10 g L^{-1}) to obtain a sodium ion concentration of 1000 mg L^{-1}, and then sized to 100 mL. The samples were detected using an atomic absorption spectrophotometer. The total K concentrations were calculated using the following formula:

$$TK = C \times \frac{100\,mL}{m} \times \frac{100\,mL}{V}$$

TK: total K concentration (g kg^{-1}); C: K concentration in pilot sample solution obtained from the standard curve (mg mL^{-1}); V: volume of pilot sample solution (mL); m: sample weight (g).

Hydrolysable N determination

In brief, approximately 1.0 g soil samples were flatted in the outer chamber of diffusion dishes, and then 1.0 g Zn-$FeSO_4$ reductants were added. The samples were mixed with 10 mL NaOH solutions (1.8 M) for 24 h at 40 ℃. The ammonia amounts absorbed by 3 mL boric acid indicators (20 g L^{-1}) in the inner chamber of diffusion dishes were titrated with 0.01 M HCl standard solutions. The hydrolysable N concentrations were calculated using the following formula:

$$HN = \frac{0.01 \times (V_0 - V) \times 14}{m} \times 1000$$

HN: hydrolyzable N concentration (mg kg^{-1}); V_0: volume of HCl standard solution for control test (mL); V: volume of HCl standard solution for sample test (mL); m: sample weight (g).

Available P determination

In brief, approximately 5 g soil samples were added with 50 mL NH_4F (0.03 M)-HCl (0.025 M) extractant in extraction bottles, shaken (160 r min^{-1}) for 5 min at room temperature, and then the samples were filtered. Approximately 5–10 mL pilot samples were transferred into 50 mL volumetric flasks, added with 5 mL boric acid solutions, diluted to approximately 20 mL using water, and then added with 1 drop of dinitrophenol indicator. The solutions were adjusted to faint yellow using NH_3 H_2O solution (2 M) and HCl solutions (2 M), added with 5 mL molybdenum antimony antichromogenic agents, and then sized to 50 mL using water. The samples remained still for 30 min at room temperature and were detected using a spectrophotometer at 700 nm. Gradients of 0, 0.1, 0.2, 0.3, 0.4, 0.5, and 0.6 mg L^{-1} P standard solutions were used to draw the standard curve. The available P concentrations were calculated using the following formula:

$$\mathrm{AP} = (\mathrm{C} - \mathrm{C}_0) \times \frac{100\,mL}{m} \times \frac{50\,mL}{V}$$

AP: available P concentration (mg kg^{-1}); C: P concentration in pilot sample solution obtained from the standard curve (μg mL^{-1}); C_0: P concentration in cobtrol solution obtained from the standard curve (μg mL^{-1}); V: volume of pilot sample solution (mL); m: sample weight (g).

Available K determination

In brief, approximately 5 g soil samples were added with 50 mL ammonium acetate solutions (1 M) in triangular bottles, shaken (150–180 r min^{-1}) for 30 min at room temperature, and then the samples were filtered. The filtrates were detected using an atomic absorption spectrophotometer. Gradients of 0, 6, 12, 18, 24, and 30 μg mL^{-1} K standard solutions were used to draw the standard curve. The available K concentrations were calculated using the following formula:

$$\mathrm{AK} = \frac{C \times V}{m}$$

AK: available K concentration (mg kg^{-1}); C: K concentration in pilot sample solution

obtained from the standard curve (μg mL^{-1}); V: volume of extractant (mL); m: sample weight (g).
